# L-Arginine-Modified Chitosan Curcumin Nanocrystals Target M1 Macrophages via CAT-2/Clathrin-Mediated Endocytosis for Mitochondrial Protection and ALI/ARDS Therapy

**DOI:** 10.3390/pharmaceutics18040425

**Published:** 2026-03-30

**Authors:** Xiaowen Yang, Shiyue Wu, Zhiya Dou, Yuxiao Dong, Jundong Dai

**Affiliations:** 1 School of Chinese Materia Medica, Beijing University of Chinese Medicine, Yang Guang South Street, Fang-Shan District, Beijing 102488, China; yxw13383446997@163.com (X.Y.); 18801216233@163.com (S.W.); 18338077442@163.com (Z.D.); dongyuxiao2024@163.com (Y.D.); 2 Department of Chinese Medicinal Pharmaceutics, Beijing University of Chinese Medicine, Yang Guang South Street, Fang-Shan District, Beijing 102488, China

**Keywords:** ALI/ARDS, L-arginine, M1 macrophages targeting, curcumin, nanocrystals, mitochondrial delivery

## Abstract

**Background:** Acute Lung Injury/Acute Respiratory Distress Syndrome (ALI/ARDS) is a fatal inflammatory disorder driven by M1 macrophages and the associated inflammatory cascade. Targeted drug delivery to these cells is a promising therapeutic strategy. **Methods:** L-arginine was conjugated to chitosan of different molecular weights. The resulting curcumin nanocrystals (Arg-CS-Cur) were characterized for conjugation efficiency, zeta potential, stability, and drug release profile. Cellular uptake mechanisms and mitochondrial targeting were investigated in lipopolysaccharide (LPS)-induced M1 macrophages using specific endocytic inhibitors and confocal microscopy. **Results:** Low-molecular-weight chitosan (MW 50 kDa) showed the highest L-Arg conjugation efficiency (22.31%). The optimized Arg-CS-Cur nanocrystals exhibited high zeta potential (±47.5 mV), excellent stability, and a superior drug release. They were internalized by M1 macrophages more efficiently than unmodified CS-Cur or free curcumin (*p* < 0.05). Uptake occurred via clathrin-mediated endocytosis (*p* < 0.001) and was mediated by CAT-2, which was highly expressed in M1 macrophages (*p* < 0.001). Arg-CS-Cur specifically targeted the mitochondria, reducing ROS and NLRP3 expression, thus inhibiting the NLRP3 inflammasome pathway (*p* < 0.001). **Conclusions:** This L-arginine-modified chitosan-based nanodelivery system synergistically exploits CAT-2 and clathrin pathways to deliver curcumin to M1 macrophage mitochondria, inhibiting the NLRP3 inflammasome. This dual-targeted strategy offers a promising approach for treating ALI/ARDS.

## 1. Introduction

Acute lung injury (ALI) is a clinically common and critical respiratory disease caused by non-cardiogenic respiratory disorder, resulting in hypoxemia and pulmonary edema [1]. Severely affected patients often progress to more serious acute respiratory distress syndrome (ARDS), eventually leading to respiratory failure or even death [2,3]. The development of ALI/ARDS involves three phases: the acute exudative phase, the proliferation phase, and the late regressive stage or fibrotic phase [4]. Alveolar macrophages (AMs) are key regulatory cells in this pathological process [5]. During the acute exudative phase, alveolar macrophages can recognize pathogen-associated molecular patterns (PAMPs) such as lipopolysaccharide (LPS), a bacterial cell wall component, and viral double-stranded RNA, as well as damage-associated molecular patterns (DAMPs) including high-mobility group box 1 (HMGB1) via pattern recognition receptors (PRRs) [6]. These macrophages then polarize toward the classically activated phenotype (M1), a process that is dependent on the NF-κB signaling pathway [7]. M1 macrophages secrete higher levels of pro-inflammatory cytokines such as interleukin-1 (IL-1), interleukin-6 (IL-6), interleukin-1β (IL-1β), and tumor necrosis factor-α (TNF-α), and chemokines like monocyte chemoattractant protein-1 (MCP-1), C-C motif chemokine ligand 8 (CCL8), and interleukin-23 (IL-23) [5,8]. These factors recruit monocytes and neutrophils, resulting in their excessive infiltration in the pulmonary parenchyma and alveolar spaces [9]. Under the influence of cytotoxic substances such as reactive oxygen species (ROS) and nitric oxide (NO) released by these cells [10], alveolar epithelial cells and vascular endothelial cells undergo damage. This subsequently induces symptoms including impaired gas exchange, pulmonary edema, and hypoxemia [11]. In the middle and late stages of the disease, M1 macrophages gradually switch to M2 macrophages, and the severity of pulmonary fibrosis is determined by the polarization balance between M1/M2 macrophages [12]. Therefore, targeted drug delivery to M1 macrophages to modulate their polarization status may serve as an effective and promising strategy for the treatment of ALI/ARDS [13].

Nanoparticle-based drug delivery systems offer several advantages for the treatment of ALI/ARDS, including targeted delivery, prolonged drug half-life, enhanced bioavailability and improved tissue penetration, thus exhibiting significant therapeutic potential [14]. Multiple studies have demonstrated that inhalable nanoparticles based on polymers, lipids, and inorganic materials have been developed for pulmonary drug delivery [15]. Yao et al. synthesized a nanoparticle Man@Cel-NPs, which was delivered via trachea and improved the retention rate of Cel-NPs at the site of inflammation by targeting mannose receptors on AMs [16]. Researchers have developed the inhalable multifunctional hybrid bionic nanoplatform D-SEL, which employs a stepwise delivery strategy for the delivery of DNase and MPS, achieving synergistic effects through dual-stage actions. This nanoplatform remodels the pulmonary immune microenvironment and promotes lung tissue repair [17]. Compared with traditional drug delivery methods such as oral and injectable administration, tracheal inhalation enables direct drug delivery to the target organ (lung), increases local drug concentration, minimizes the toxic side effects caused by systemic drug distribution [18], enhances patient compliance, and improves clinical efficacy [19].

The research team previously developed a cationic amino acid-modified nanoliposome, Arg-Cur-Lip, using the thin-film hydration method. Evaluations of its cellular uptake, both in vitro and in vivo, showed that the L-arginine (L-Arg) surface modification enhanced targeted delivery to lung tissues and M1 macrophages upon inhalation, highlighting the therapeutic potential of L-Arg-modified nanocarriers for ALI/ARDS [20]. To overcome the limitations of liposomes, such as their poor stability and low drug-loading capacity, we synthesized L-Arg-modified chitosan (CS) by the 1-(3-Dimethylaminopropyl)-3-ethylcarbodiimide hydrochloride (EDC) and N-Hydroxysuccinimide (NHS) coupling method for preparing curcumin nanocrystals (Arg-CS-Cur). The resulting nanocrystals exhibited a significantly higher drug-loading capacity of 78.93 ± 2.51% compared to that of liposomes (3.23 ± 0.05%). Following intratracheal nebulization in a rat model of ALI, Arg-CS-Cur exhibited excellent lung selectivity and targeting capability. Pharmacodynamic analyses revealed that Arg-CS-Cur significantly reduced the secretion of key M1 macrophage-derived proinflammatory factors, including NO, IL-6, and TNF-α. These findings suggested that L-Arg-functionalized nanocrystals could be preferentially internalized by M1 macrophages [21]. This may be attributed to the tight correlation between macrophage polarization and L-Arg metabolism [22]. Specifically, during polarization, inducible nitric oxide synthase (iNOS) and arginase 1 (Arg-1) catalyze the degradation of L-Arg, acting as key effector molecules in both M1 and M2 macrophages [23]. Macrophages express transport systems that mediate L-Arg uptake, with cationic amino acid transporters (CAT1, -2, or -3) considered primary uptake pathways [24]. Studies confirm that compared with CAT-1, LPS-induced M1 macrophages exhibit significantly upregulated expression of CAT-2B [25]. Administration of acute inflammation inhibitors to LPS-induced macrophages markedly reduces intracellular levels of iNOS and CAT-2B expression [26]. Based on these findings, it is reasonable to propose that CAT-2B plays a more critical role in the targeted delivery of Arg-Cur-Lip and Arg-CS-Cur to M1 macrophages. Consequently, the L-arginine-modified nanodrugs can be specifically and efficiently internalized into M1 macrophages from the extracellular environment.

Furthermore, numerous studies have demonstrated that macrophage polarization and mitochondrial metabolism exhibit direct or indirect crosstalk [27,28]. Mitochondrial dysfunction within M1 macrophages impairs their repolarization toward anti-inflammatory M2 macrophages. This indicates that, at the cellular level, mitochondria are also served as a key target for regulating macrophage phenotype [29]. Mitochondria possess a double-membrane structure, with the inner membrane further invaginates to form cristae that divide the mitochondrion into distinct compartments [30]. The complex interplay between phospholipids on these membranes and cristae architecture poses a barrier, limiting drug access to targets within the mitochondrial matrix [31]. However, as the site of the tricarboxylic acid (TCA) cycle and electron transport chain (ETC) activity, mitochondria exhibit a negative membrane potential relative to other organelles. Highly positively charged nanoparticles can escape via the “sponge effect”, avoiding complete degradation by the acidic environment and enzymes [32], and bind more readily to mitochondrial membranes through electrostatic interactions [33]. For instance, triphenylphosphonium (TPP) is a lipophilic cationic small molecule that targets mitochondria. Its ability to enter mitochondria, driven by the transmembrane potential, has led to its widespread application in mitochondrial-targeted nanodelivery systems [34]. Recent studies have also utilized peptide backbones containing cationic and hydrophobic amino acid residues to achieve precise mitochondrial targeting [35]. Building on this, if L-Arg-modified nanomedicines can achieve mitochondrial-specific delivery within M1 macrophages, it would significantly enhance their therapeutic efficacy and advantages in treating pulmonary inflammation.

Preliminary results confirmed the superior therapeutic efficacy of Arg-CS-Cur over unmodified curcumin, though its modification conditions and targeting mechanisms remain to be fully explored. This study optimized Arg-CS preparation through the EDC-NHS coupling method, investigating how chitosan molecular weight affects the L-Arg conjugation efficiency, resulting nanoparticle size, zeta potential, and subsequent uptake efficiency by M1 macrophages. Based on these results, chitosan with a molecular weight of 50 kDa was selected to prepare Arg-CS and labeled with fluorescein isothiocyanate (FITC). With 1,1′-dioctadecyl-3,3,3′,3′-tetramethylindotricarbocyanine iodide (DiR) as the fluorescent probe, FITC-Arg-CS-Cur-DiR hybrid nanocrystals were prepared. The targeted uptake mechanism was investigated using an in vitro M1 macrophage model, with an in-depth investigation of the pivotal role of cationic amino acid transporter (CAT) in targeted uptake. Additionally, differences in mitochondrial targeting efficiency within cells via distinct uptake pathways were compared. This study aims to provide novel new insights into the precise delivery of nanomedicines at the mechanistic level. It is expected to provide a reference and theoretical support for the targeted treatment of acute inflammatory lung diseases such as ALI/ARDS, thereby facilitating the clinical translation of nanomedicines in the treatment of pulmonary inflammatory disorders.

## 2. Materials and Methods

### 2.1. Materials

Curcumin (Cur), 2-(N-morpholino)ethanesulfonic acid (MES) buffer, regenerated cellulose dialysis bags (3500 Da), Ethylisopropylamiloride (EIPA), Methyl-β-cyclodextrin (M-β-CD), Chlorpromazine (CPZ), and Asymmetric dimethylarginine (ADMA) were purchased from Shanghai Yuanye Bio-Technology Co., Ltd. (Shanghai, China); Chitosan samples of different molecular weights (MW; 50, 100, and 200 kDa), all with a degree of deacetylation (DD) of 90%, were purchased from Shanghai Acmec Biochemical Technology Co., Ltd. (Shanghai, China); L-arginine was purchased from Beijing Coolaber Technology Co., Ltd. (Beijing, China); EDC and NHS were purchased from Tokyo Chemical Industry Co., Ltd. (Tokyo, Japan); High-glucose Dulbecco’s modified eagle medium (DMEM) and phosphate-buffered saline (PBS) were purchased from Gibco Inc. (Waltham, MA, USA); Australian fetal bovine serum (FBS), Penicillin-Streptomycin Solution were purchased from Corning Inc. (Corning, NY, USA); LPS, interleukin-4 (IL-4), and dimethyl sulfoxide (DMSO) were purchased from Sigma-Aldrich (St. Louis, MO, USA); Cell Counting Kit-8 (CCK-8), ROS Assay Kit for Superoxide Anion with Dihydroethidium (DHE), Mito-Tracker Red, Hoechst 33342 and DAPI were purchased from Beyotime Biotech Inc. (Shanghai, China); RNA Extraction Kits, Evo M-MLV RT Mix Kits, SYBR Green Premix Pro Taq HS qPCR Kits were purchased from Hunan Accurate Bioengineering Co., Ltd. (Changsha, China); DNA Isolation Mini Kit was purchased from Vazyme Biotech Co., Ltd. (Nanjing, China); Digitoxin, HEPES-KOH buffer were purchased from Yeasen Biotechnology Co., Ltd. (Shanghai, China); Radioimmunoprecipitation assay (RIPA) protein lysis buffer, loading buffer, BCA protein quantification kit, and primary antibodies Anti-STING, Anti-p-STING, Anti-NLRP3 were purchased from Cell Signaling Technology, Inc. (Danvers, MA, USA); Nitrocellulose membranes and ECL chemiluminescent substrate were purchased from Merck Millipore (Burlington, MA, USA); Bovine serum albumin (BSA) standard was purchased from Merck KGaA (Darmstadt, Germany); Primary antibody Anti-CAT-2 was purchased from Proteintech Group, Inc. (Rosemont, IL, USA); Goat anti-Rabbit IgG-HRP was purchased from Jackson ImmunoResearch Laboratories, Inc. (West Grove, PA, USA).

### 2.2. Methods

#### 2.2.1. Cell Culture

The murine RAW264.7 macrophage cell line was obtained from the Benna Cell Bank. Cells were maintained in the high-glucose DMEM supplemented with 10% Australian fetal bovine serum and 1% penicillin-streptomycin at 37 °C in a humidified 5% CO_2_ incubator.

LPS (1 μg·mL^−1^) and IL-4 (20 ng·mL^−1^) were used to induce RAW264.7 cells to differentiate into M1 and M2 macrophages, respectively. The Macropinocytosis inhibitor EIPA (50 μmol·L^−1^), the clathrin-mediated endocytosis inhibitor CPZ (10 μg·mL^−1^), the caveolae-mediated endocytosis inhibitor M-β-CD (10 mg·mL^−1^), and ADMA (10 μg·mL^−1^), a competitive substrate inhibitor of CAT-mediated transport, were used to inhibit different uptake pathways.

#### 2.2.2. Synthesis and Characterization of Different Molecular Weight Chitosans Modified with L-Arginine

We synthesized arginine-modified chitosan (Arg-CS) using the EDC-NHS coupling method. Three distinct Arg-CS were prepared based on chitosan molecular weight: MW 50 kDa (Arg-CS-1), MW 100 kDa (Arg-CS-2), and MW 200 kDa (Arg-CS-3). For each synthesis, chitosan (1 g) was dissolved in MES buffer (pH 5.5). Predetermined amounts of L-arginine (1.944 g), EDC (3.208 g), and NHS (1.929 g) (corresponding to a molar ratio of 1.0:1.5:1.5) were dissolved in MES buffer, and the carboxyl groups of L-arginine were activated via incubation of the mixture in an ice-water bath for 2 h. The chitosan solution was mixed with the activated arginine solution and stirred magnetically for 48 h at room temperature. Upon completion, the pH was adjusted to 8.0 to quench the reaction. The products were dialyzed against distilled water for 48 h using a dialysis membrane with a molecular weight cutoff (MWCO) of 3500 Da and then freeze-dried to obtain Arg-CS.

The successful conjugation of L-arginine to chitosan was verified by Fourier-transform infrared (FT-IR) spectroscopy (EQUINOX 55, Bruker, Karlsruhe, Germany) and proton nuclear magnetic resonance (^1^H NMR) spectroscopy (600 MHz, Bruker, Karlsruhe, Germany). An elemental analyzer (Unicube, Elementar, Langenselbold, Germany) was employed to determine the carbon (C) and nitrogen (N) content, enabling calculation of the degree of substitution (DS) of L-Arg in Arg-CS.

#### 2.2.3. Preparation of Curcumin Nanocrystals Using Different Molecular Weight Chitosans Modified with L-Arginine

To compare with arginine-modified chitosan curcumin nanocrystals (Arg-CS-Cur), chitosan curcumin nanocrystals (CS-Cur) were prepared in parallel using chitosan of three different molecular weights (MW 50 kDa, MW 100 kDa, MW 200 kDa). Curcumin was dissolved in ethanol and mixed with acetic acid solutions of CS or Arg-CS. The mixture was homogenized using a 200 W ultrasonic probe in the ice-water bath to obtain a suspension containing curcumin nanocrystals. An initial low-speed centrifugation step (3000 rpm for 10 min) was performed to remove large aggregates. Then the supernatant was collected and centrifuged at a higher speed (12,000 rpm for 15 min) to pellet the nanocrystals. The precipitates obtained were CS-Cur nanocrystals (CS-Cur-1, CS-Cur-2, CS-Cur-3) and Arg-CS-Cur nanocrystals (Arg-CS-Cur-1, Arg-CS-Cur-2, Arg-CS-Cur-3), corresponding to the three molecular weights of chitosan/Arg-CS respectively.

#### 2.2.4. Characterization of Curcumin Nanocrystals Using Different Molecular Weight Chitosans Modified with L-Arginine

The CS-Cur and Arg-CS-Cur suspensions were diluted, deposited onto 200-mesh copper grids, air-dried, and observed under a transmission electron microscope (JEM 2100Plus, JEOL, Akishima, Japan). The nanocrystal particle size and zeta potential were determined using a dynamic light scattering particle size analyzer (Zetasizer Nano-ZS90, Malvern, Malvern, UK). The size stability was assessed by monitoring changes in the particle size distribution over 48 h, with measurements taken at 0, 6, 24, 36, and 48-h time points. Curcumin nanocrystals were precisely weighed, dissolved in ethanol by sonication to a fixed volume, and the curcumin content was quantified to calculate the drug loading capacity of the nanocrystals according to Equation (1) [21].

(1) $$\mathrm{Drug}\;\mathrm{Loading}\;(\mathrm{DL}\%)=\frac{\mathrm{Mass}\;\mathrm{of}\;\mathrm{the}\;\mathrm{loaded}\;\mathrm{drug}}{\mathrm{Total}\;\mathrm{mass}\;\mathrm{of}\;\mathrm{the}\;\mathrm{drug}\;\mathrm{delivery}\;\mathrm{system}}\times 100\%$$

To simulate the pulmonary microenvironment, the release profiles of CS-Cur and Arg-CS-Cur nanocrystals were evaluated under two distinct conditions. To mimic pulmonary surfactant, nanocrystal suspensions were incubated with a pre-warmed (37 °C) 0.2% Tween-80 aqueous solution in a constant-temperature shaker, and samples were collected at 5, 10, 20, 30, and 45 min. In parallel, to simulate pulmonary interstitial tissue fluid, aliquots of the nanocrystals were incubated in pre-warmed (37 °C) serum-free high-glucose DMEM, with sampling performed at 0, 2, 4, 6, and 8 h. All collected samples were centrifuged at 5000 rpm for 10 min. The curcumin concentration in the supernatant was quantified, and the cumulative drug release was calculated relative to the initial drug loading amount. The resulting release data were fitted to well-established kinetic models to elucidate the underlying release mechanisms.

#### 2.2.5. In Vitro Cytotoxicity

The in vitro cytotoxicity of curcumin, CS-Cur, and Arg-CS-Cur was evaluated using the CCK-8 assay. RAW264.7 cells were evenly seeded into 96-well plates at a density of 2 × 10^4^ cells per well. Wells containing only medium served as the blank control, while cells cultured in drug-free medium served as the control group. Serum-free cell culture medium containing curcumin, CS-Cur, or Arg-CS-Cur (at curcumin equivalent concentrations of 10 μM, 20 μM, 30 μM, 40 μM, 50 μM, and 60 μM) was prepared using serum-free medium for cell culture. After 24 h of incubation, the supernatant was carefully removed. Subsequently, 100 μL of prewarmed serum-free medium containing 10% CCK-8 reagent was added to each well. The plates were incubated in the cell culture incubator for 2 h. A multi-mode microplate reader (SpectraMax i3x, Molecular Devices, San Jose, CA, USA) was used to measure the absorbance at 450 nm, and the cell viability was calculated relative to that of the control group.

#### 2.2.6. In Vitro Targeting Efficiency Evaluation

The effect of chitosan molecular weight on the targeted uptake of Arg-CS-Cur nanocrystals by M1 macrophages was evaluated qualitatively and quantitatively using confocal laser scanning microscopy (CLSM) and flow cytometry (FCM). M1 macrophages were induced from RAW264.7 cells by LPS stimulation to establish the cell model. The cells were then incubated for 6 h in serum-free medium supplemented with curcumin, CS-Cur, or Arg-CS-Cur nanocrystals (At a curcumin equivalent concentration of 20 μM). Following incubation, the cells were washed three times with PBS (3 min per wash) and fixed with 4% paraformaldehyde (PFA) for 30 min at room temperature, followed by staining with 100 μL DAPI solution for 15 min in the dark. The stained samples were observed under a confocal laser scanning microscope (FV3000, Olympus Corporation, Tokyo, Japan).

M1 macrophages were incubated with each formulation for 6 h. After washing three times with ice-cold PBS, the cells were harvested, resuspended in 500 μL of PBS, filtered through a 300-mesh cell strainer, and transferred into flow cytometry tubes. The intracellular fluorescence intensity was detected using a flow cytometer (CytoFLEX, Beckman Coulter, Brea, CA, USA) to compare the cellular uptake levels of curcumin, CS-Cur, and Arg-CS-Cur nanocrystals by M1 macrophages.

#### 2.2.7. Preparation and Characterization of FITC-Arg-CS-DiR Hybrid Nanocrystals

To investigate the mechanism of targeted uptake of Arg-CS-Cur by M1 macrophages, FITC and DiR fluorescent probes were used to label the L-arginine-modified chitosan shell and the drug-loaded nanocrystals, respectively.

Arg-CS (MW 50 kDa) was dissolved in 0.1 M acetic acid to prepare a 2.0 mg·mL^−1^ solution and reacted with 10 mL of FITC methanol solution (2.0 mg·mL^−1^) with magnetic stirring in the dark for 3 h at room temperature. The FITC-Arg-CS was then precipitated by adjusting the pH to 8.0, collected by centrifugation (1000 rpm for 15 min), and washed several times with 70% methanol solution until no fluorescence was detected in the supernatant using a fluorescence spectrophotometer (λ_Ex_/λ_Em_ = 495/520 nm). The precipitate was subsequently resuspended in 10 mL of 0.1 mol·L^−1^ acetic acid. Under light-protected conditions, dialysis was performed using a dialysis membrane with a MWCO of 3500 Da for 72 h to obtain FITC-Arg-CS. The successful labeling of the FITC was confirmed by FT-IR spectroscopy.

FITC-Arg-CS was split into two portions for the preparation of two types of nanocrystals. To one portion, 0.75 mL of curcumin ethanol solution (2 mg·mL^−1^) was added dropwise. To the other portion, a mixture of 0.75 mL of the same curcumin solution and 0.2 mL of DiR ethanol solution (1 mg·mL^−1^) was added dropwise. Both groups were subjected to probe sonication (200 W, 20 min) in an ice-water bath and centrifuged at 3000 rpm for 10 min. Then the supernatants were further centrifuged at 12,000 rpm for 15 min to obtain FITC-Arg-CS-Cur and FITC-Arg-CS-Cur-DiR nanocrystals.

Arg-CS (MW 50 kDa) was dissolved in 0.1 M acetic acid. 0.75 mL of curcumin ethanol solution (2 mg·mL^−1^) and 0.2 mL of DiR ethanol solution (1 mg·mL^−1^) were added dropwise. The mixture was subjected to probe sonication (200 W, 20 min) in an ice-water bath and centrifuged at 3000 rpm for 10 min. Then the supernatants were centrifuged at 12,000 rpm for 15 min to obtain Arg-CS-Cur-DiR nanocrystals.

The particle size and zeta potential of the nanocrystals (FITC-Arg-CS-Cur, Arg-CS-Cur-DiR, and FITC-Arg-CS-Cur-DiR) were characterized by dynamic light scattering (DLS). The nanocrystals were stored in the dark at 4 °C, and their particle sizes were measured at 0, 6, 24, 36, and 48-h time points to assess particle size stability.

#### 2.2.8. In Vitro Cellular Uptake Mechanism

M1 macrophages were co-incubated with serum-free medium containing FITC-Arg-CS-Cur-DiR (20 μM, curcumin equivalent concentration) for 6 h. Cells were then washed three times with ice-cold PBS, fixed with 4% PFA for 30 min at room temperature, washed three times again with ice-cold PBS, and stained with 100 μL DAPI for 15 min in the dark. Cellular uptake was visualized by CLSM.

Different pathway inhibitors, along with serum-free medium containing Arg-CS-Cur-DiR, were added separately to M1 macrophages. After incubation for 2 h, cells were washed three times with cold PBS. They were then resuspended in 500 μL of PBS, filtered through a 300-mesh cell strainer, and transferred into flow cytometry tubes. The intracellular DiR fluorescence intensity was analyzed by FCM.

#### 2.2.9. Mitochondrial Targeting Analysis

M1 macrophages were incubated with serum-free medium containing Arg-CS-Cur (20 μM, curcumin equivalent concentration) and different pathway inhibitors for 6 h. The cells were then stained with Hoechst 33342 and Mito-tracker Red (200 nM) for 30 min in the dark. After washing three times with prewarmed DMEM, the cells were imaged by CLSM to analyze the colocalization of between mitochondria and nanocrystals.

#### 2.2.10. Intracellular ROS Detection

The accumulation of ROS was quantified via DHE staining. LPS-induced M1 macrophages were treated with serum-free medium containing Arg-CS-Cur (20 μM, curcumin equivalent concentration) and different pathway inhibitors for 6 h. Then the cells were resuspended in 0.1% DHE staining solution and incubated at 37 °C in a cell culture incubator in the dark for 30 min. After washing with ice-cold PBS, the DHE fluorescence intensity was analyzed by FCM.

#### 2.2.11. Cellular DNA Isolation

Serum-free medium containing Arg-CS-Cur (20 μM, curcumin equivalent concentration) and different pathway inhibitors were added to M1 macrophages, followed by incubated for 6 h. After washing with ice-cold PBS, the cells were harvested and divided into two equal aliquots. For cytoplasmic DNA extraction, one aliquot was centrifuged to obtain a pellet, which was then permeabilized with 250 μL of digitonin buffer (25 μg·mL^−1^ in HEPES-KOH) for 10 min. After the centrifugation at 800 rpm for 10 min to remove cell debris, the supernatant was subjected to high-speed centrifugation at 14,000 rpm for 10 min [36]. The final supernatant served as the sample for cytoplasmic DNA extraction. Total genomic DNA was extracted from the other aliquot according to the protocol of the FastPure Cell/Tissue DNA Isolation Mini Kit.

#### 2.2.12. Real-Time Fluorescent Quantitative Polymerase Chain Reaction

Macrophages seeded in 6-well plates (6 × 10^5^ cells per well) and induced to the M1 phenotype were treated for 6 h in serum-free medium supplemented with Arg-CS-Cur (20 μM, curcumin equivalent concentration) and different pathway inhibitors. Total mRNA was extracted using the SteadyPure Universal RNA Extraction Kit, immediately reverse transcribed into cDNA with the Evo M-MLV RT Premix, and subjected to quantitative real-time PCR (qPCR) analysis using the SYBR Green Premix Pro TaqHS qPCR Kit III. The expression levels of Caspase-1 and IL-1β genes were normalized to glyceraldehyde-3-phosphate dehydrogenase (GAPDH). Cytosolic mitochondrial DNA (mtDNA) levels were quantified by qPCR and normalized to ribosomal RNA 18S (Rn18s). The primer sequences used for PCR amplification are shown in Table 1.

#### 2.2.13. Western Blot Analysis

RIPA lysis buffer containing protease inhibitors was added to cells after induction and drug administration. The lysates were collected and centrifuged to extract the supernatant. Protein concentrations were quantified by the BCA assay. Equal protein amounts (30 μg per lane) were resolved by sodium dodecyl sulfate-polyacrylamide gel electrophoresis (SDS-PAGE), transferred to a polyvinylidene fluoride (PVDF) membrane, and blocked. The membranes were then probed overnight at 4 °C with primary antibodies against CAT-2, STING, Phospho-STING, and NLRP3 (diluted in 3% BSA-TBST). The membrane was incubated with HRP-conjugated secondary antibody (diluted in 3% BSA-TBST) for incubation at room temperature for 60 min. After washing, the protein bands were visualized using enhanced chemiluminescence (ECL) reagent, and the band intensities were quantified using ImageJ 1.53 software.

#### 2.2.14. Statistical Analysis

All experiments were performed in triplicate, and data presented as mean ± standard deviation (SD). Statistical analyses were performed using Graphpad Prism 9.0 software. For comparisons between two groups, Student’s *t*-test was employed. Comparisons among multiple groups were performed using one-way analysis of variance (ANOVA). Differences were considered statistically significant if *p* < 0.05 and highly significant if *p* < 0.01.

## 3. Results

### 3.1. Characterization of Arg-CS

Preliminary studies have demonstrated that Arg-CS-Cur nanocrystals exhibit excellent targeting capability toward M1 macrophages, enhancing drug concentration in the lungs and thereby potentiating anti-inflammatory effects. As a common drug carrier, chitosan has a molecular weight that significantly influences its solubility, solution viscosity, and drug release characteristics, making it a crucial parameter for evaluation [37]. To further improve the targeting capability of Arg-CS-Cur nanocrystals, three chitosans with distinct molecular weights were selected. This design aimed to analyze the influence of chitosan molecular weight on the physicochemical properties of the nanocrystals and their uptake efficiency by M1 macrophages.

**Figure 1:**
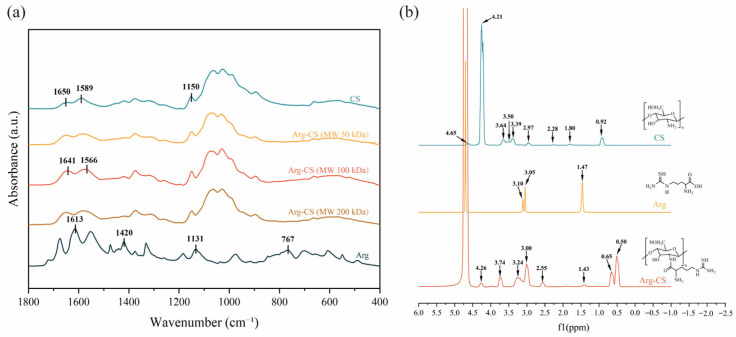
Characterization of Arg-CS. (**a**) FTIR spectra of arginine, chitosan, and Arg-CS. (**b**) ^1^H NMR spectra of arginine, chitosan, and Arg-CS.

FT-IR analysis revealed significant differences between Arg-CS and L-arginine/chitosan. The absorption peaks at 1641 cm^−1^ and 1566 cm^−1^ were assigned to the N–H deformation vibrations of amide I and amide II, respectively [38,39]. The characteristic primary amine bending vibration peak at 1589 cm^−1^ in the original chitosan disappeared after arginine modification and was replaced by a newly formed amide II band [40]. This indicated the formation of amide bonds in the product after the reaction, demonstrating that the α-carboxyl group of L-arginine had bonded with the amino groups on chitosan [41]. Comparison with the ^1^H NMR spectra of chitosan and L-arginine revealed that changes in the signal peaks at 1.43 ppm and 4.26 ppm in the spectrum of the newly synthesized Arg-CS indicated successful grafting of L-arginine onto the chitosan terminus. The disappearance of the terminal group peak of glucosamine, along with the splitting and enhancement of the protonated amino group signal, further confirmed alterations in the side-chain structure. These results collectively demonstrated the successful synthesis of Arg-CS from L-arginine and chitosan. FT-IR, proton nuclear magnetic resonance (^1^H NMR), and X-ray photoelectron spectroscopy (XPS) are currently the most commonly employed techniques. The primary objective of such characterization is to confirm the successful formation of amide bonds between chitosan and L-Arg. These techniques can provide information about the functional groups present in the sample. Luo et al. also modified low-molecular-weight chitosan with L-Arg via the EDC-NHS coupling method. Compared with the spectrum of CS, the Arg-CS spectrum exhibited three bands at 1670 cm^−1^, 1540 cm^−1^, and 1290 cm^−1^, which were assigned to the characteristic amide I, II, and III bands. This observation indicated the successful covalent linkage between Arg and CS [42]. These results are shown in Figure 1.

The carbon and nitrogen contents of Arg-CS prepared from chitosan with different molecular weights were determined. The DS of arginine onto chitosan was calculated using a formula that effectively eliminates interference from other elements [43]. The results indicated that under identical reaction and dialysis conditions, the DS gradually decreased (22.31%, 18.44%, 14.03%) with increasing molecular weight of chitosan (MW 50 kDa, MW 100 kDa, MW 200 kDa), demonstrating that Arg-CS synthesized from low-molecular-weight chitosan achieved the highest modification efficiency. When chitosan with MW 50 kDa was used and the reaction time was extended to 36, 48, and 60 h, the DS values were 17.52%, 22.31%, and 26.32%, indicating that the DS increased with reaction time. Under fixed chitosan molecular weight and reaction time, dialysis for 24, 48, and 60 h resulted in DS values of 23.30%, 22.31%, and 21.60%. Prolonged dialysis slightly diminished the substitution efficiency. Considering both experimental duration (i.e., reaction and dialysis times) and DS variation, the optimal conditions were determined as a reaction time of 48 h and a dialysis time of 48 h.

### 3.2. Characterization of Arg-CS-Cur

**Figure 2:**
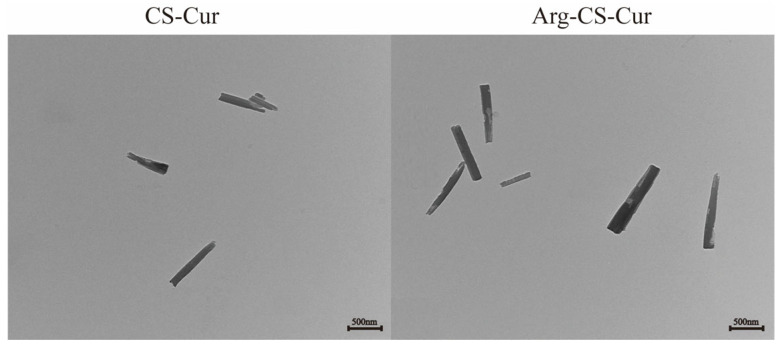
Microstructure of CS-Cur and Arg-CS-Cur under transmission electron microscopy (×6000).

Transmission electron microscopy images (Figure 2) revealed rod-like nanocrystals with lengths ranging from approximately 400 nm to 600 nm. The morphology of the nanocrystals showed no significant changes following L-arginine modification, confirming that the modification process did not alter their structure.

**Figure 3:**
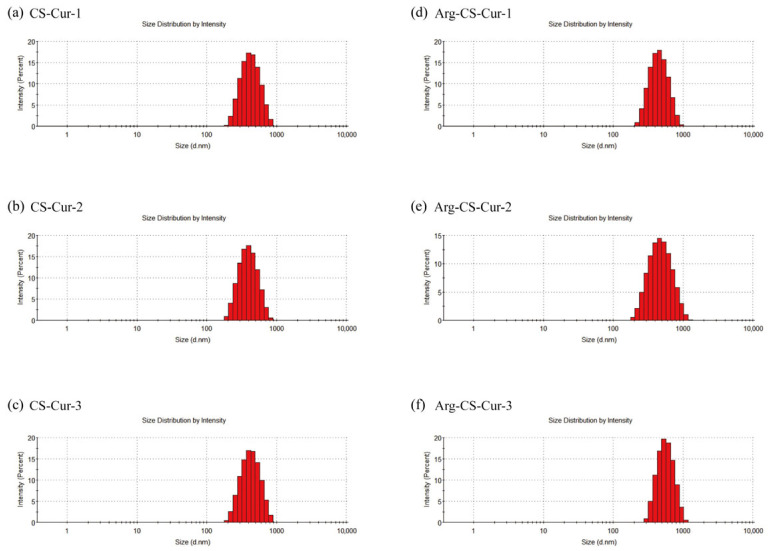
Particle size distribution of CS-Cur (**a**–**c**) and Arg-CS-Cur (**d**–**f**). 1, 2, and 3 represent different molecular weights of chitosan (MW 50 kDa, MW 100 kDa, and MW 200 kDa).

Under the same experimental conditions, the particle size, PDI, and zeta potential of the nanocrystals were shown in Table 2. The results indicated that the nanoparticle size distribution exhibited a symmetric unimodal normal distribution and low dispersion, suggesting that the nanoparticle sizes were relatively consistent (Figure 3).

**Figure 4:**
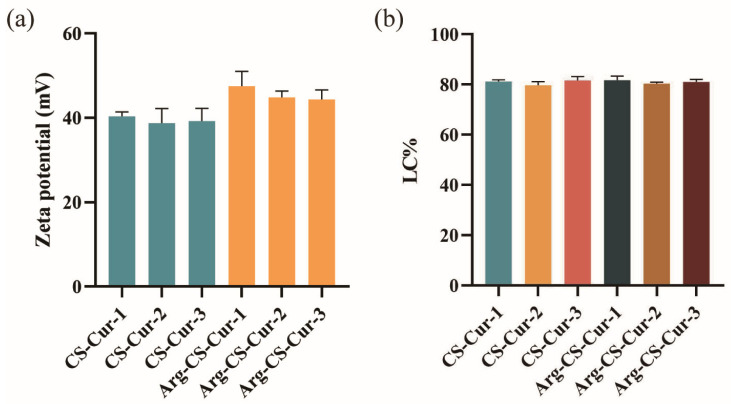
Zeta potential (**a**), and drug loading (**b**) of CS-Cur and Arg-CS-Cur. 1, 2, and 3 represent different molecular weights of chitosan (MW 50 kDa, MW 100 kDa, and MW 200 kDa). Data are expressed as mean ± SD (*n* = 3).

Zeta potential serves as a measure of surface charge, where a higher absolute surface charge value generally corresponds to better particle stability [44]. It depends on the DD of chitosan, as DD governs the charge density along the polymer backbone [45,46]. Typically, a higher zeta potential value indicates greater particle stability because the increased surface charge enhances interparticle repulsion and reduces aggregation. The Arg-CS-Cur group exhibited a higher zeta potential, suggesting that L-Arg modification enhanced the stability of nanocrystals in the aqueous medium. The Arg-CS-Cur modified with chitosan of MW 50 kDa demonstrated the highest zeta potential, consistent with its performance in subsequent stability experiments. Drug loading (DL) capacities of CS-Cur and Arg-CS-Cur were determined. The DL values for the CS-Cur group were (81.2 ± 0.7)%, (79.7 ± 1.5)%, and (81.6 ± 1.6)%, respectively, while those for Arg-CS-Cur were (81.7 ± 1.6)%, (80.4 ± 0.5)%, and (81.0 ± 1.0)%. This demonstrated that chitosan molecular weight and L-Arg modification did not significantly influence the drug loading capacity of the prepared curcumin nanocrystals. (Figure 4).

**Figure 5:**
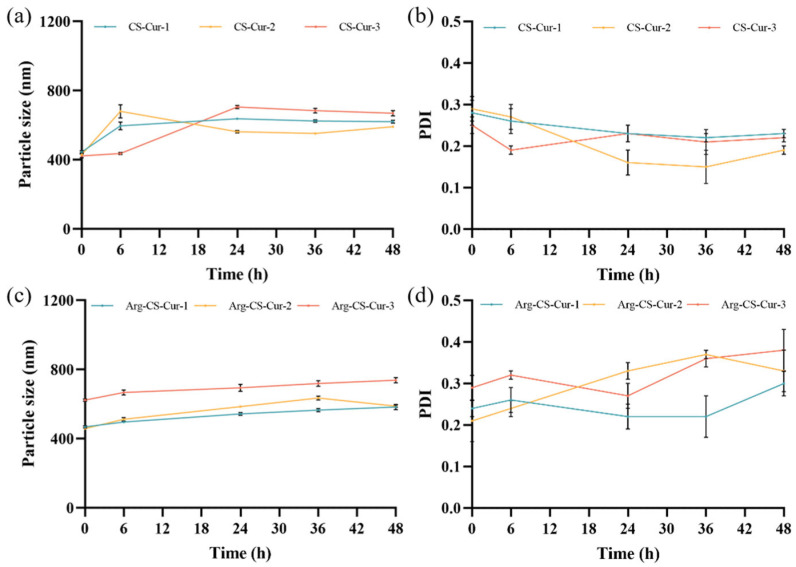
Particle size stability of CS-Cur (**a**) and Arg-CS-Cur (**c**), and PDI of CS-Cur (**b**) and Arg-CS-Cur (**d**). 1, 2, and 3 represent different molecular weights of chitosan (MW 50 kDa, MW 100 kDa, and MW 200 kDa). Data are expressed as mean ± SD (*n* = 3).

Throughout the 48-h observation period, both CS-Cur and Arg-CS-Cur maintained stable particle sizes (Figure 5). The PDI of CS-Cur remained consistently below 0.3 at all time points. For Arg-CS-Cur, PDI values were generally slightly higher than those of CS-Cur, remaining within the range of 0.2–0.4 throughout the study. This moderate increase is likely attributable to the cationic nature of the arginine modification, which may promote mild surface interactions or reorganization in the storage medium rather than indicating significant aggregation. Some fluctuations were observed, particularly for Arg-CS-Cur-2 and Arg-CS-Cur-3 at later time points, yet all values stayed below 0.4, which is considered acceptable for nanoparticle dispersions in biological contexts [47]. The samples maintained uniform color and distribution without significant changes. Notably, compared to unmodified chitosan, the arginine-modified samples demonstrated higher stability throughout the experiment, exhibiting a positive correlation between particle size and molecular weight. Based on the results, the stability of curcumin nanocrystals prepared from chitosan of MW 50 kDa modified with L-Arg is optimal.

**Figure 6:**
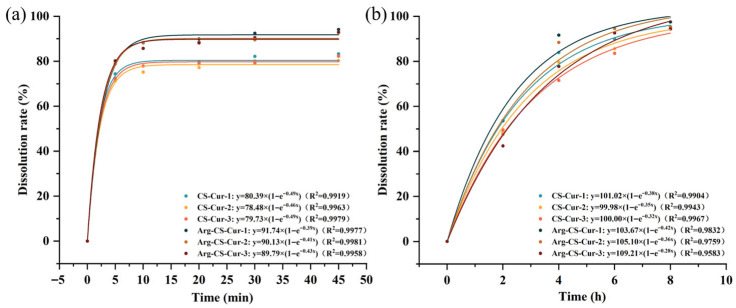
Release profiles and first-order kinetic model fitting of CS-Cur and Arg-CS-Cur release profiles in 0.2% Tween-80 aqueous solution (**a**) and serum-free medium (**b**). 1, 2, and 3 represent different molecular weights of chitosan (MW 50 kDa, MW 100 kDa, and MW 200 kDa). Data are expressed as mean ± SD (*n* = 3).

The in vitro release profiles of CS-Cur and Arg-CS-Cur nanocrystals prepared with different molecular weights of chitosan (50 kDa, 100 kDa, and 200 kDa) were evaluated in two release media: 0.2% Tween-80 aqueous solution and serum-free high-glucose DMEM, as shown in Figure 6. In both media, nanocrystals prepared with low-molecular-weight chitosan (50 kDa) showed the highest cumulative release, reaching 83.09% for CS-Cur-1 and 94.03% for Arg-CS-Cur-1 in Tween-80 medium, indicating that the molecular weight of chitosan is a significant factor influencing the release of curcumin nanocrystals. This may be attributed to the smaller particle size of nanocrystals prepared from low-molecular-weight chitosan, which exhibits a higher specific surface area, facilitating drug dissolution and diffusion. Additionally, its superior water solubility allows the long chains in the molecule to swell and extend more readily in water, promoting permeation of the dissolution medium and drug release [48,49]. Furthermore, Arg-CS-Cur nanocrystals generally showed higher release rates than unmodified CS-Cur nanocrystals. This is attributed to arginine’s excellent water solubility. The guanidino group of arginine (pKa ≈ 12.48) confers a positive charge in neutral, acidic, or alkaline environments [50]. The arginine modification enhanced the hydrophilicity of Arg-CS-Cur nanocrystals, increasing their interaction with aqueous media. It may also have created intermolecular voids between chitosan chains via electrostatic repulsion, promoting polymer swelling and thereby facilitating curcumin release. Compared to 0.2% Tween-80 aqueous solution, the nanocrystals exhibited more sustained-release profiles in DMEM. Release was nearly linear from 2 to 4 h, gradually slowed from 4 to 6 h, and was essentially complete by 8 h. The release rate of Arg-CS-Cur remained consistently higher than that of CS-Cur in DMEM, consistent with the trend observed in the 0.2% Tween-80 aqueous solution release medium.

Fitting of the in vitro release profiles to kinetic models demonstrated that the release behavior of the nanocrystals in both media conformed to the first-order kinetic model (R^2^ > 0.95). This suggested that release was primarily diffusion-driven, influenced mainly by the concentration gradient formed after curcumin dissolution and the permeability of the chitosan coating on the nanocrystal surface. In high-glucose DMEM, the drug release initially exhibited a near-linear increase. Over time, the release rate gradually approached a steady state. This indicated that the initial release was primarily driven by diffusion via the concentration gradient. As the release proceeded, the concentration gradient decreased, leading to a reduction in the diffusion rate and ultimately resulting in a dynamic equilibrium.

The release rate constants (*k*) derived from the first-order model provided further support for this mechanism. In DMEM, a clear molecular weight dependence was observed: as chitosan molecular weight decreased from 200 kDa to 50 kDa, the *k* value increased progressively for both CS-Cur (from 0.32 to 0.38) and Arg-CS-Cur (from 0.28 to 0.42). This trend was consistent with the smaller particle size and larger specific surface area of low-molecular-weight chitosan nanocrystals. These structural characteristics facilitated faster drug diffusion in media where polymer swelling governed the release process. In Tween-80, this molecular weight trend was less pronounced, as drug release was driven by surfactant-mediated solubilization rather than polymer swelling. Under these conditions, factors such as drug–matrix affinity and matrix tortuosity played a more dominant role, overshadowing the effect of particle size [51].

In Tween-80 solution, Arg-CS-Cur exhibited a lower *k* value than unmodified CS-Cur. This resulted from the electrostatic repulsion between guanidino groups, which introduced a more tortuous diffusion pathway. Additionally, hydrogen bonding between arginine and curcumin enhanced drug–matrix affinity. These factors hindered surfactant-mediated extraction of curcumin, leading to a slower release rate. In DMEM, where release was governed by medium penetration and polymer swelling, the hydrophilicity and positive charge of arginine promoted water uptake and chain relaxation, facilitating drug diffusion and giving Arg-CS-Cur-1 the highest release rate among all formulations.

The results of the above characterization indicators collectively indicated that chitosan molecular weight and chemical modification were key factors influencing the physicochemical properties and release behavior of Arg-CS-Cur nanocrystals. Low-molecular-weight chitosan (50 kDa) consistently yielded smaller particle sizes, higher zeta potential, and enhanced colloidal stability, while arginine modification further improved surface charge and hydrophilicity. These characteristics facilitated the adhesion of nanocrystals to cell surfaces via electrostatic interactions, thereby enhancing the endocytic efficiency of macrophages.

In vitro release studies revealed distinct release behaviors of the nanocrystals in different simulated media. In Tween-80 solution, which mimics the surfactant-rich environment of the alveolar surface, arginine-modified nanocrystals exhibited relatively lower release constants (*k*) compared to unmodified formulations. In contrast, in DMEM, which simulates interstitial fluid and intracellular environments, the release constants (*k*) of arginine-modified nanocrystals were higher. This differential release profile suggested that Arg-CS-Cur may remain relatively stable at the alveolar surface while enabling more efficient drug release upon cellular internalization or within the tissue environment. Importantly, in vitro release conditions do not fully recapitulate the complex in vivo environment. In the presence of cells, the release rate of nanocrystals would be reduced, and nanocrystals could be internalized via phagocytosis prior to substantial extracellular drug release. This mechanism was supported by preliminary in vivo findings from our group [21], which demonstrated that Arg-CS-Cur efficiently targeted pulmonary macrophages and exerted functional effects, providing direct evidence for its in vivo efficacy.

### 3.3. In Vitro Targeting of Arg-CS-Cur

**Figure 7:**
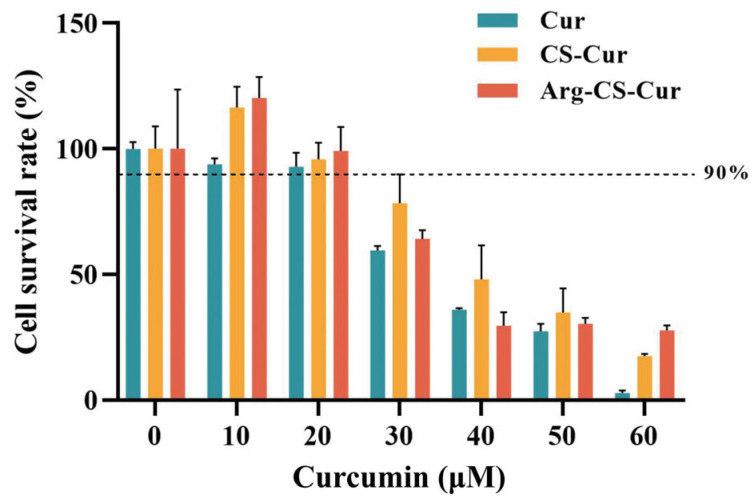
Effects of curcumin, CS-Cur, and Arg-CS-Cur on the viability of RAW 264.7 cells. Data are expressed as mean ± SD (*n* = 3).

The cytotoxicity of curcumin, CS-Cur, and Arg-CS-Cur against RAW264.7 cells was assessed using the CCK-8 assay (Figure 7). At curcumin concentrations below 20 μM, none of the nanocrystals showed significant cytotoxicity after 24-h incubation. Moreover, CS-Cur and Arg-CS-Cur promoted a certain degree of cell proliferation. At curcumin concentrations ≥30 μM, while the cytotoxicity of all groups increased in a dose-dependent manner, the modified nanocrystals exhibited lower cytotoxicity than curcumin and demonstrated higher biocompatibility with RAW264.7 cells [20,52]. This protective effect may be attributed potentially due to the cell-protective effects of chitosan [53,54].

**Figure 8:**
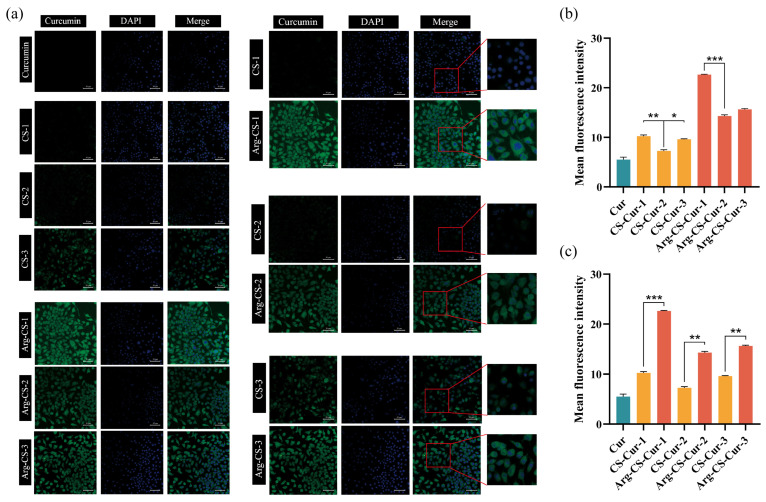
In Vitro Targeting of Arg-CS-Cur. (**a**) LSCM images of different groups co-incubated with M1 macrophages (×40). Scale bar = 50 µm. (**b**,**c**) Quantitative analysis of the intracellular fluorescence intensity from (**a**). 1, 2, and 3 represent different molecular weights of chitosan (MW 50 kDa, MW 100 kDa, and MW 200 kDa). Data are expressed as mean ± SD (*n* = 3). *** *p* < 0.001, ** *p* < 0.01 and * *p* < 0.05.

Using M1 macrophages as a model, we evaluated the in vitro targeting ability of curcumin, CS-Cur, and Arg-CS-Cur using CLSM. Results indicated that after incubation of M1 macrophages with medium containing curcumin, CS-Cur, or Arg-CS-Cur for 6 h, L-Arg-modified nanocrystals showed significant in vitro targeting ability to M1 macrophages, with a higher uptake efficiency than that of CS-Cur and curcumin (*p* < 0.01). Arg-CS-Cur prepared from chitosan of MW 50 kDa exhibited the highest fluorescence intensity. The CS-Cur group showed a consistent trend in fluorescence intensity with the Arg-CS-Cur group. The results are presented in Figure 8.

**Figure 9:**
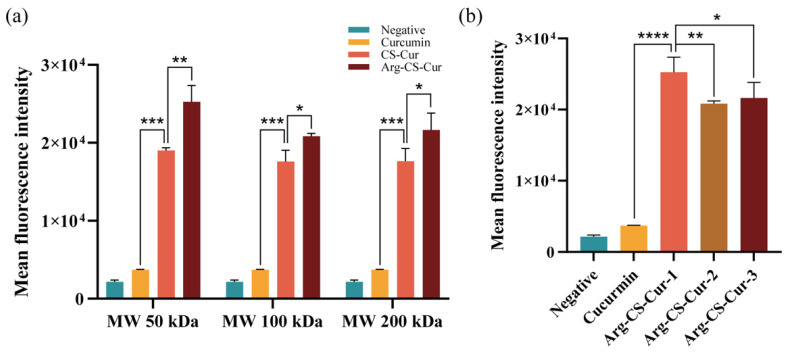
Flow cytometric analysis of fluorescence intensity within M1 macrophages. (**a**) Fluorescence intensity in the CS-Cur and Arg-CS-Cur groups; (**b**) Comparison among Arg-CS-Cur groups with different molecular weights. 1, 2, and 3 represent different molecular weights of chitosan (MW 50 kDa, MW 100 kDa, and MW 200 kDa). Data are expressed as mean ± SD (*n* = 3). **** *p* < 0.0001, *** *p* < 0.001, ** *p* < 0.01 and * *p* < 0.05.

Flow cytometry was employed to quantitatively analyze the phagocytic capacity of M1 macrophages for each group (Figure 9). The fluorescence intensity was significantly higher in the Arg-CS-Cur group than in the unmodified CS-Cur group (*p* < 0.05), with both groups showing markedly higher fluorescence intensity than the curcumin-only control group (*p* < 0.001). Within the CS-Cur groups, the CS-Cur group prepared from chitosan with an MW 50 kDa exhibited a slightly higher fluorescence intensity than the other two groups, though this difference was not statistically significant. In contrast, the Arg-CS-Cur group prepared from chitosan with an MW 50 kDa showed a marked increase in fluorescence intensity, which was statistically significant compared to the other molecular weight groups (*p* < 0.05). These results were consistent with the findings of CLSM, both indicating that the Arg-CS modification enhanced the uptake of curcumin nanocrystals by M1 macrophage. Arg-CS-Cur showed excellent in vitro targeting capability to M1 macrophages, and chitosan with a low molecular weight resulted in the optimal uptake efficiency.

Collectively, these results suggest that both the molecular weight of chitosan and the modification of chitosan with L-arginine influenced the cellular uptake efficiency of curcumin. The biological activity of chitosan is influenced by its molecular weight and degree of deacetylation [55]. Low-molecular-weight chitosan exhibits superior water solubility and biodegradability [56], facilitating easier interaction with receptors on cell membranes, thereby enhancing drug adhesion to and uptake by cell membranes [57,58]. Miao et al. reported that using liposomes modified with chitosan oligosaccharides improved the delivery efficiency of paclitaxel, indicating that low-molecular-weight chitosan oligosaccharides could overcome the limitations associated with high-molecular-weight chitosan as a drug carrier, such as poor solubility and a tendency to aggregate. This modification strategy increased drug accumulation in the lungs, confirming its effectiveness as a drug delivery vehicle [59]. L-Arg, as a basic amino acid, bears a positive charge under normal physiological conditions. After modifying inherently positively charged chitosan with L-arginine, the positive charge density on the surface of the nanocarrier is further increased. This promotes stronger electrostatic interactions with the negatively charged phospholipids on cell membranes, enhancing the adhesion and uptake of the nanocarriers by the cells [60,61].

### 3.4. Characterization of FITC-Arg-CS-Cur-DiR

FITC is a fluorescent dye commonly used in biological and chemical research, with high hydrophilicity and intense green fluorescence. Its isothiocyanate group (R-N=C=S) readily reacts with the primary amine groups (-NH_2_) of chitosan, forming FITC-labeled chitosan through the formation of a thiourea bond [62]. DiR is a lipophilic near-infrared fluorescent dye that can penetrate deep tissues [63]. It can be physically encapsulated into nanocrystals via the solvent–nonsolvent method, enabling fluorescent localization and in vivo/in vitro tracking [64].

**Figure 10:**
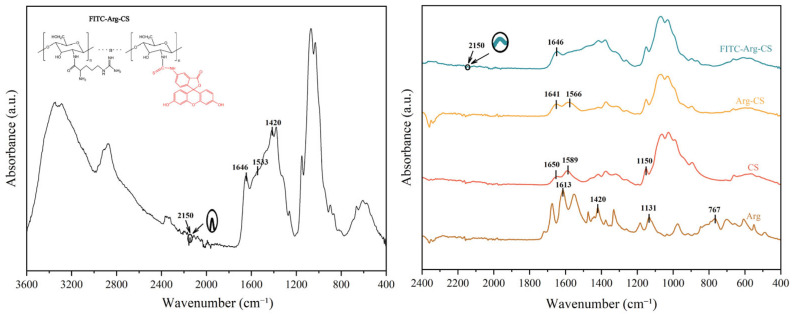
FTIR spectra of arginine, chitosan, Arg-CS, and FITC-Arg-CS.

The infrared spectrum provided direct visual evidence for the chemical structural changes following FITC labeling of Arg-CS (Figure 10). The most prominent feature was the appearance of a new absorption peak at 2150 cm^−1^, which was attributed to the C=N stretching vibration characteristic of the isothiocyanate group (N=C=S) in the FITC molecule [65]. However, the consumption of the N=C=S group during the reaction attenuated the intensity of this peak. Additionally, a newly emerging peak at 1533 cm^−1^ was assigned to the C=C stretching vibration of the benzene ring in FITC [66]. Another new peak that appeared at 1420 cm^−1^ may be associated with the specific C-H bending vibration of FITC.

**Figure 11:**
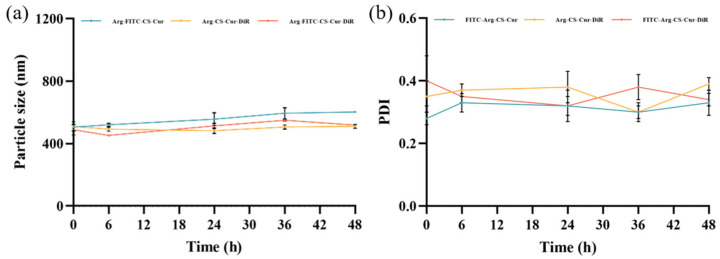
Particle size stability (**a**) and PDI (**b**) of FITC-Arg-CS-Cur, Arg-CS-Cur-DiR, and FITC-Arg-CS-Cur-DiR. Data are expressed as mean ± SD (*n* = 3).

The particle size and zeta potential results are presented in Table 3. The results indicated that FITC labeling or DiR hybridization did not significantly alter the nanocrystal size and uniformity, as evidenced by the stable particle size and consistently similar PDI values of all three formulations throughout the 48-h observation period. No significant changes in color or distribution were observed for FITC-Arg-CS-Cur, Arg-CS-Cur-DiR, and FITC-Arg-CS-Cur-DiR, nor did any aggregation or precipitation occur. These results demonstrated that the nanocrystals maintained good particle size stability for up to 48 h after preparation (Figure 11).

### 3.5. Targeted Uptake Mechanisms of FITC-Arg-CS-Cur-DiR

In the aforementioned experiment, FITC and DiR were used to label Arg-CS and the drug, respectively. To investigate the targeted uptake mechanism of Arg-CS-Cur by M1 macrophages, fluorescence colocalization analysis was used to qualitatively determine whether the nanocrystals are taken up by M1 macrophages as intact particles or as released free drug. Meanwhile, flow cytometry and Western blot experiments were applied to quantify the uptake efficiency via different pathways.

CLSM revealed the uptake of nanocrystals by M1 macrophages. Green fluorescence from FITC and red fluorescence from DiR were both uniformly distributed within the cells. In the merged image, the high degree of colocalization was visualized as yellow or orange signals. This indicated that FITC-labeled FITC-Arg-CS and DiR-hybridized nanocrystals were co-localized intracellularly, suggesting that the FITC-Arg-CS-Cur-DiR hybrid nanocrystals were predominantly taken up by M1 macrophages as an integrated structure.

**Figure 12:**
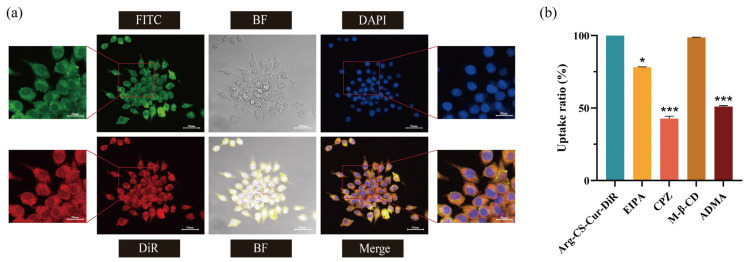
(**a**) CLSM images of FITC-Arg-CS-Cur-DiR co-incubated with M1 macrophages (×40). Scale bar = 50 µm. (**b**) Cellular uptake efficiency of Arg-CS-Cur-DiR under treatment with different endocytosis inhibitors. Data are expressed as mean ± SD (*n* = 3). Statistical significance was calculated versus the Arg-CS-Cur-DiR group. *** *p* < 0.001 and * *p* < 0.05.

Flow cytometry results following treatment with different pathway inhibitors showed that CPZ significantly inhibited the uptake of nanocrystals by M1 macrophages, with an inhibition rate of 57.26% (*p* < 0.001). Treatment with ADMA also led to a significant reduction in drug uptake, showing an inhibition rate of 48.99% (*p* < 0.001). In contrast, EIPA treatment slightly reduced drug uptake (*p* < 0.05). M-β-CD had almost no effect on drug uptake. These findings indicated that clathrin-mediated endocytosis was the predominant pathway for nanocrystal uptake by M1 macrophages, whereas macropinocytosis contributed to the process but was not the dominant mechanism, and caveolin-mediated endocytosis did not serve as a primary route. Furthermore, the CAT protein was clearly involved in mediating drug transport and significantly enhanced cellular drug uptake efficiency. All of these results are summarized in Figure 12.

**Figure 13:**
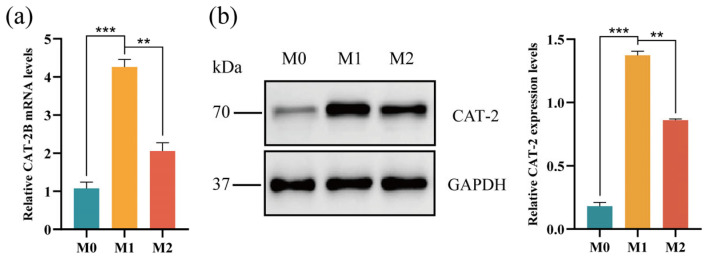
(**a**) CAT-2B gene expression levels in M0, M1, and M2 macrophages. (**b**) CAT-2 protein expression levels in M0, M1, and M2 macrophages. Data are expressed as mean ± SD (*n* = 3). *** *p* < 0.001 and ** *p* < 0.01.

CAT-2B gene expression levels were examined in M0, M1, and M2 macrophages (Figure 13). Significant differences in CAT-2B gene expression were observed among RAW264.7 cells (M0), LPS-induced M1 macrophages, and IL-4-induced M2 macrophages. In M1 macrophages, the mRNA level of the Slc7a2 gene, which encodes the CAT-2B protein, was significantly higher than that in M0 (*p* < 0.001) and M2 (*p* < 0.01) macrophages, confirming that LPS-induced M1 macrophages upregulated CAT-2B gene expression. Concurrently, CAT-2 protein expression results also matched the gene expression findings. Combined with the observation that M1 macrophages exhibited significantly higher uptake capacity for Arg-CS-Cur nanocrystals than M0 and M2 macrophages, these results further validated the pivotal role of the CAT protein in the targeted uptake of Arg-CS-Cur nanocrystals by M1 macrophages.

### 3.6. Mitochondrial Targeting of Arg-CS-Cur

Inflammation is typically triggered by PRRs expressed on cells, which can recognize PAMPs derived from viruses and bacteria, as well as endogenous DAMPs. This recognition elicits immune responses to combat infection and facilitate tissue repair [67]. Certain mitochondrial components and metabolites can act as DAMPs that are released into the cytoplasm or extracellular environment, thereby promoting inflammatory responses [68]. Accumulating evidence has demonstrated that mitochondrial dysfunction is one of the pathogenic mechanisms underlying ALI/ARDS [69,70]. Therefore, targeted drug delivery to mitochondria for modulating associated inflammatory signaling pathways has emerged as a promising therapeutic strategy. While our previous work has verified the cellular uptake mechanism of Arg-CS-Cur, its intracellular pharmacological mechanism remains to be fully elucidated. To further elucidate the uptake pathway of Arg-CS-Cur nanocrystals and their influence on intracellular delivery, we examined the intracellular colocalization of Arg-CS-Cur with mitochondria and key molecules in associated inflammatory pathways.

**Figure 14:**
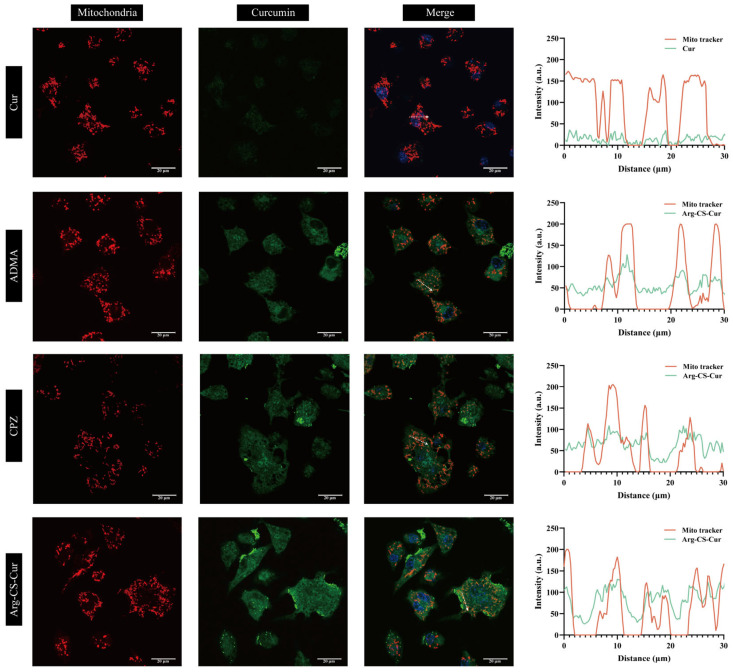
Colocalization of Arg-CS-Cur with mitochondria observed by CLSM.

**Figure 15:**
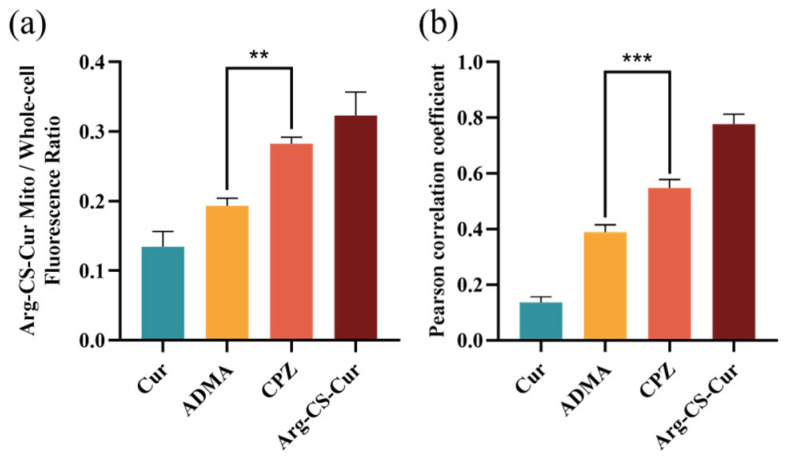
Quantitative analysis of mitochondrial localization of Arg-CS-Cur. (**a**) Fluorescence ratio of Arg-CS-Cur in mitochondria. The fluorescence ratio was calculated as (integrated fluorescence intensity in mitochondria)/(total cellular fluorescence intensity). (**b**) Pearson’s colocalization coefficient for Arg-CS-Cur and mitochondria. Data are expressed as mean ± SD (*n* = 3). *** *p* < 0.001 and ** *p* < 0.01.

CLSM was used to analyze the intracellular distribution and mitochondrial colocalization of Cur, Arg-CS-Cur, and Arg-CS-Cur after cellular uptake, following inhibition of the CAT protein or clathrin-mediated endocytosis by ADMA or CPZ (see Figure 14 and Figure 15). When M1 macrophages were treated with curcumin alone, the fluorescence signal was weak. The fluorescence ratio, which was calculated as the ratio of Arg-CS-Cur fluorescence in mitochondria to total cellular fluorescence, was the lowest among all groups (0.1346), indicating poor cellular uptake and minimal mitochondrial accumulation of free curcumin. Consistently, the Pearson’s colocalization coefficient was also low (R = 0.1361), further confirming the lack of mitochondrial targeting capacity of unmodified curcumin. In contrast, Arg-CS-Cur exhibited a significantly higher fluorescence ratio (0.3232), demonstrating that L-arginine modification substantially enhanced mitochondrial accumulation. This finding was corroborated by a high colocalization coefficient (R = 0.7770), indicating close spatial association between the nanocrystals and mitochondria. The mitochondrial accumulation efficiency of Arg-CS-Cur in M1 macrophages (∼32%) was within a range comparable to that achieved by triphenylphosphonium (TPP), a well-established mitochondrial targeting moiety, which was reported to localize to mitochondria at efficiencies of 40–45% in cells [71]. This further highlighted the potential of this modification strategy.

Further analysis revealed that the subsequent mitochondrial localization of the nanocrystals was modulated by different uptake mechanisms. Following CAT protein inhibition, the fluorescence ratio decreased to 0.1932, whereas inhibition of clathrin-mediated endocytosis resulted in a value of 0.2824. The reduction in the fluorescence ratio was significantly greater in the ADMA-treated group compared to the CPZ-treated group (*p* < 0.01), suggesting that CAT-mediated transport played a more prominent role in directing nanocrystals to mitochondria. This conclusion was supported by the colocalization coefficient results. In the ADMA-treated group, the coefficient decreased to 0.3898, which was significantly lower than that in the CPZ-treated group (0.5467, *p* < 0.001), further confirming the key contribution of the CAT pathway to mitochondrial targeting.

Based on these findings, we hypothesized that when the dominant clathrin-mediated endocytic pathway was inhibited, the CAT protein cooperated with alternative uptake mechanisms to facilitate nanocrystal delivery to mitochondria, thereby enhancing mitochondrial targeting.

Mitochondrial dysfunction triggers inflammatory responses primarily through two key signaling cascades. The first involves mtDNA activating cyclic GMP-AMP synthase (cGAS), which transmits signals to the stimulator of interferon genes (STING), thereby initiating a downstream signaling cascade that drives inflammatory responses [72]. The second pathway is driven by mtDNA and ROS, which induce the activation of the NLR family pyrin domain containing 3 (NLRP3) inflammasome, leading to the secretion of inflammatory cytokines and subsequent inflammatory responses [73].

**Figure 16:**
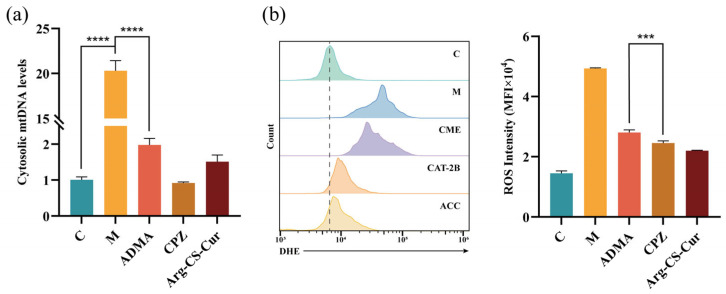
(**a**) Cytosolic mitochondrial DNA level. (**b**) Fluorescence intensity of ROS. Data are expressed as mean ± SD (*n* = 3). **** *p* < 0.0001 and *** *p* < 0.001.

**Figure 17:**
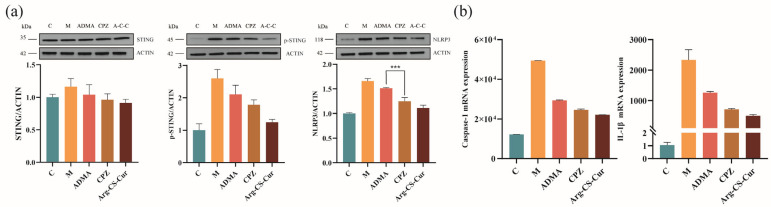
(**a**) Protein expression levels of STING, p-STING, and NLRP3. (**b**) mRNA expression levels of Caspase-1 and IL-1β. Data are expressed as mean ± SD (*n* = 3). *** *p* < 0.001.

mtDNA leakage and ROS levels are critical indicators for assessing mitochondrial function [74]. As shown in Figure 16, compared with the control group, M1 macrophages exhibited significantly elevated cytoplasmic mtDNA leakage and ROS levels, indicating severe mitochondrial dysfunction. Treatment with Arg-CS-Cur significantly reduced both indices (*p* < 0.0001), demonstrating its efficacy in alleviating mitochondrial damage. Under conditions of inhibited different uptake pathways, mtDNA leakage and ROS levels in the ADMA-treated group (CAT-inhibited) were higher than those in the CPZ-treated group, suggesting that CAT-mediated uptake enhances the mitochondrial targeting and therapeutic efficacy of the nanocrystals. Western blot and RT-qPCR analysis (Figure 17) revealed that the expression changes of mitochondrial damage-associated inflammatory signaling molecules (STING, NLRP3, Caspase-1, IL-1β) followed the same trend as the aforementioned indicators, further supporting that the CAT protein facilitates nanocrystal targeting to mitochondria.

Additionally, the results showed that during inflammation, both the cGAS-STING and NLRP3 inflammasome pathways were activated upon stimulation by mtDNA and ROS stimulation. Arg-CS-Cur treatment downregulated the expression of key molecules in these pathways. Although cytosolic mtDNA levels and STING protein expression exhibited trends consistent with the aforementioned indices, no statistically significant differences were observed between the ADMA-treated and CPZ-treated groups. Conversely, ROS levels and NLRP3 expression differed significantly between the two groups (*p* < 0.001). This discrepancy may be attributed to the preferential accumulation of nanocrystals at mitochondrial sites with mtDNA and active ROS generation via CAT-mediated uptake, thereby enhancing its inhibitory effects on ROS and NLRP3. In contrast, the targeting of Arg-CS-Cur to cytosolic mtDNA appears less precise than its targeting to mitochondrial loci. Even after cellular entry, the inhibitory effect of the nanocrystals on this pathway remained relatively modest.

Collectively, these findings demonstrated that Arg-CS-Cur is primarily internalized via CAT protein-mediated uptake, subsequently exerting significant mitochondrial targeting. This results in the inhibition of key signaling molecules in mitochondrial-associated inflammatory pathways, effectively attenuating the inflammatory response. Although clathrin-mediated endocytosis is the dominant uptake route, its inhibition is compensated for by the CAT protein-facilitated upregulation of alternative endocytic pathways, such as macropinocytosis or caveolin-dependent endocytosis. This compensatory mechanism maintains intracellular delivery and mitochondrial targeting efficiency of the nanocrystals. Consequently, the CPZ-treated group exhibited more profound attenuation of inflammation than the ADMA-treated group.

## 4. Discussion

The cellular uptake pathway of nanoparticles determines their delivery efficiency and intracellular distribution, which is essential for achieving the intended therapeutic effect. Nanoparticle properties, such as size, shape, and stiffness, influence the selection of uptake pathways [75]. In addition, electrostatic interactions represent another key factor. Compared to unmodified, uncharged nanoparticles, positive or negative charge modifications may enhance cellular uptake efficiency [76]. In this study, Arg-CS was synthesized via the EDC-NHS coupling method using chitosan of varying molecular weights. The results demonstrated that the modification efficiency of chitosan with L-arginine followed the order: MW 50 kDa > MW 100 kDa > MW 200 kDa, with the molecular weight of 50 kDa exhibiting the highest arginine modification rate (22.31%). Furthermore, macrophage internalization of nanocrystals prepared from different molecular weight chitosan followed the order: MW 50 kDa > MW 200 kDa > MW 100 kDa. These findings suggest that nanocrystals derived from lower molecular weight chitosan are internalized more efficiently. This phenomenon can be explained by the more effective modification of low-molecular-weight chitosan by arginine, which imparts a higher positive surface charge to the nanocrystals, a conclusion consistent with prior research [21].

The common mechanisms for nanoparticle entry into cells are primarily categorized into macropinocytosis and receptor-mediated endocytosis (RME). Macropinocytosis is initiated by local actin polymerization, which drives the formation of membrane protrusions that form macropinosomes; these subsequently transport their contents via the lysosomal system [77]. RME encompasses several subtypes, with clathrin-mediated endocytosis (CME) and caveolin-mediated endocytosis (CVME) being the most relevant to nanoparticle uptake. In CME, clathrin present on the cell surface forms coated pits that engulf target molecules, which are then internalized as intracellular vesicles. These vesicles ultimately mature into endosomes and fuse with lysosomes [78]. Caveolae are flask-shaped invaginations of the cell membrane. Caveolin participates in CVME by forming caveolin-coated vesicles upon cargo uptake, which subsequently fuse with early endosomes [79].

CAT protein-mediated recognition and adsorption may be a prerequisite for the efficient uptake of cationic amino acid-modified drugs by M1 macrophages. Due to the negatively charged lipid bilayer on the cell surface, cationic nanoparticles are more readily targeted and penetrate the cell membrane [80]. Negatively charged nanoparticles are typically internalized via the CVME pathway, whereas positively charged nanoparticles preferentially enter cells through the CME pathway [81,82]. L-arginine modification enabled Arg-CS-Cur to be specifically recognized by the cationic amino acid transporter CAT on the surface of M1 macrophages. This not only enhanced Arg-CS-Cur enrichment at the cell membrane, but also facilitated synergistic uptake via the clathrin-mediated endocytic pathway. Inhibitor experiments revealed that blocking clathrin-mediated endocytosis (CPZ) and CAT protein function (ADMA) reduced the uptake rates to 57.26% and 48.99%, respectively. This indicated that both pathways play critical roles in the cellular uptake of Arg-CS-Cur. These findings clarify that L-arginine modification enables cell-specific targeting and efficient uptake via the clathrin-mediated endocytosis pathway with CAT involvement, establishing a delivery foundation for precisely regulating NLRP3-associated inflammatory functions mediated by mitochondria in M1 macrophages.

Having established its cellular targeting specificity, we further investigated the intracellular targets and mechanisms of action of this nanodrug. The colocalization of Arg-CS-Cur with mitochondria demonstrated that nanocrystals internalized via CAT protein-mediated uptake exhibited superior mitochondrial targeting, a finding subsequently corroborated by pharmacological efficacy assays. This observation suggested that when the dominant clathrin-mediated endocytic pathway is inhibited, the specific adsorption mediated by the CAT protein may trigger compensatory upregulation of alternative uptake pathways such as macropinocytosis. These alternative pathways might be more conducive to the delivery of the drug to the mitochondrial compartment [83]. Examination of the two major mitochondrial-associated inflammatory pathways further revealed that Arg-CS-Cur exerted a more pronounced effect on the NLRP3 inflammasome pathway. This differential impact may stem from its preferential action at mitochondrial sites where mtDNA and ROS are generated, whereas its intervention in the cytosolic regions is relatively limited. This also provides additional evidence for the higher mitochondrial targeting efficacy of the nanocrystals.

In this study, chitosan was employed as a carrier and modified with arginine to prepare drug nanocrystals. Compared with other nanocarriers [84], this synthetic approach is relatively straightforward and the nanocrystalline structure enables a high drug loading capacity. To endow chitosan carriers with targeting capability, strategies reported in the literature often involve ligand modifications such as hyaluronic acid or charge-based adsorption, yet these approaches frequently lack pathophysiological specificity [85,86]. In contrast to such strategies, the present study revealed that CAT protein expression on the surface of M1 macrophages is upregulated after polarization. Therefore, by enhancing the modification efficiency of L-arginine, Arg-CS-Cur achieved improved targeting toward M1 macrophages, enabling inflammation-responsive precision delivery.

In addition to achieving targeting at the cellular level, a further focus of this study was to facilitate drug delivery to mitochondria. Conventional mitochondrial targeting strategies represented by triphenylphosphonium (TPP) relied on electrostatic interactions to accumulate within mitochondria [87]. However, such mitochondrion-targeting moieties inherently lack cell-type selectivity; their preferential accumulation in cancer cells often results from upregulated mitochondrial activity and more negative membrane potential [88]. In comparison, Arg-CS-Cur nanocrystals not only achieved selective recognition of M1 macrophages through the CAT-mediated transport pathway but also enhanced mitochondrial targeting, thereby expanding the scope of mitochondrial targeting from non-selective accumulation to dual cellular and organelle targeting.

The synthesis of Arg-CS-Cur utilized L-arginine and chitosan, both of which are naturally derived, offering lower cost and better biocompatibility compared to synthetic nanomaterials [89]. Through arginine modification, this system extended the application of chitosan-based carriers from the cellular level to the organelle level while endowing mitochondrial targeting strategies with cell selectivity. This design concept takes advantage of the upregulated expression of an endogenous transporter under inflammatory conditions and may offer a new avenue for the targeted treatment of inflammatory diseases.

Building upon previous research, enhanced the modification rate of L-arginine and optimized the preparation process of Arg-CS-Cur, leading to improved targeting efficiency of the nanocrystals. The underlying targeting mechanism was elucidated as uptake by M1 macrophages via the CAT protein-mediated clathrin-mediated endocytosis pathway. Further investigation revealed that this specific uptake route enabled more precise action at mitochondrial sites within the cells. In summary, this study highlights the unique value of cationic amino acid modification strategies, exemplified by L-arginine, in drug delivery. This study holds potential for precisely regulating M1 macrophage polarization through the targeted treatment of mitochondrial dysfunction, thereby providing a novel theoretical and practical foundation for the clinical management of ALI/ARDS.

## Figures and Tables

**Table 1:** Sequences of Primers for RT-qPCR.

Target		Sequence (5′-3′)
mtNd1	Forward	TTACTTCTGCCAGCCTGACCCAT
	Reverse	TCTGATTCTCCTTCTGTCAGGTCG
Rn18s	Forward	TAAGCTTGCGTTGATTAAGTCCC
	Reverse	GGGCCTCACTAAACCATCCAA
Slc7a2	Forward	GGAGGATGGGTTGCTTTTCAA
	Reverse	CGAGGGCCTTCAGGTCAAAA
Casp1	Forward	TGCTTTCTGCTCTTCAACACCA
	Reverse	CCAAGTCACAAGACCAGGCATAT
IL-1β	Forward	AATGAAAGACGGCACACCCA
	Reverse	ACTCCACTTTGCTCTTGACTTCT
GAPDH	Forward	TGTGTCCGTCGTGGATCTGA
	Reverse	TTGCTGTTGAAGTCGCAGGAG

**Table 2:** Particle size, PDI, and Zeta Potential of Nanocrystals (Mean ± SD, *n* = 3).

Nanocrystals	Particle Size (nm)	PDI	Zeta Potential (mV)
CS-Cur (MW 50 kDa)	445.4 ± 6.4	0.281 ± 0.028	40.4 ± 1.0
CS-Cur (MW 100 kDa)	430.8 ± 4.4	0.290 ± 0.032	38.8 ± 3.4
CS-Cur (MW 200 kDa)	422.3 ± 11.7	0.245 ± 0.016	39.3 ± 3.0
Arg-CS-Cur (MW 50 kDa)	468.6 ± 5.7	0.240 ± 0.002	47.5 ± 3.5
Arg-CS-Cur (MW 100 kDa)	458.1 ± 2.5	0.208 ± 0.039	44.9 ± 1.5
Arg-CS-Cur (MW 200 kDa)	456.0 ± 11.1	0.232 ± 0.021	44.4 ± 2.2

**Table 3:** Particle size, PDI, and Zeta Potential of Hybrid Nanocrystals (Mean ± SD, *n* = 3).

Hybrid Nanocrystals	Particle Size (nm)	PDI	Zeta Potential (mV)
FITC-Arg-CS-Cur	505.1 ± 5.9	0.275 ± 0.021	26.2 ± 1.3
Arg-CS-Cur-DiR	510.0 ± 29.3	0.350 ± 0.048	36.5 ± 2.6
FITC-Arg-CS-Cur-DiR	488.0 ± 33.5	0.397 ± 0.075	22.4 ± 2.4

## Data Availability

The datasets generated during and/or analysed during the current study are available from the corresponding author on reasonable request.

## Abbreviations

The following abbreviations are used in this manuscript:

ALI	Acute lung injury
ARDS	Acute respiratory distress syndrome
AMs	Alveolar macrophages
PAMPs	Pathogen-associated molecular patterns
LPS	Lipopolysaccharide
DAMPs	Damage-associated molecular patterns
HMGB1	High-mobility group box 1
PRRs	Pattern recognition receptors
IL-1	Interleukin-1
IL-6	Interleukin-6
IL-1β	Interleukin-1β
TNF-α	Tumor necrosis factor-α
MCP-1	Monocyte chemoattractant protein-1
CCL8	C-C motif chemokine ligand 8
IL-23	Interleukin-23
ROS	Reactive oxygen species
NO	Nitric oxide
L-Arg	L-arginine
CS	Chitosan
EDC	1-(3-Dimethylaminopropyl)-3-ethylcarbodiimide hydrochloride
NHS	N-Hydroxysuccinimide
iNOS	Inducible nitric oxide synthase
Arg-1	Arginase 1
CAT	Cationic amino acid transporter
TCA	Tricarboxylic acid
ETC	Electron transport chain
TPP	Triphenylphosphonium
FITC	Fluorescein isothiocyanate
DiR	1,1′-dioctadecyl-3,3,3′,3′-tetramethylindotricarbocyanine iodide
Cur	Curcumin
MES	2-(N-morpholino)ethanesulfonic acid
EIPA	Ethylisopropylamiloride
M-β-CD	Methyl-β-cyclodextrin
CPZ	Chlorpromazine
ADMA	Asymmetric dimethylarginine
MW	Molecular weights
DD	Degree of deacetylation
DMEM	Dulbecco’s modified eagle medium
PBS	Phosphate-buffered saline
FBS	Fetal bovine serum
IL-4	Interleukin-4
DMSO	Dimethyl sulfoxide
CCK-8	Cell Counting Kit-8
RIPA	Radioimmunoprecipitation assay
BSA	Bovine serum albumin
Arg-CS	Arginine-modified chitosan
MWCO	Molecular weight cutoff
FT-IR	Fourier-transform infrared
DS	Degree of substitution
Arg-CS-Cur	Arginine-modified chitosan curcumin nanocrystals
CS-Cur	Chitosan curcumin nanocrystals
CLSM	Confocal laser scanning microscopy
FCM	Flow cytometry
PFA	Paraformaldehyde
DHE	Dihydroethidium
RT-qPCR	Real-time fluorescent quantitative polymerase chain reaction
qPCR	Quantitative real-time quantitative real-time PCR
GAPDH	Glyceraldehyde-3-phosphate dehydrogenase
mtDNA	Mitochondrial DNA
SDS-PAGE	Sodium dodecyl sulfate-polyacrylamide gel electrophoresis
PVDF	Polyvinylidene fluoride
ECL	Enhanced chemiluminescence
SD	Standard deviation
DL	Drug loading
cGAS	Cyclic GMP-AMP synthase
STING	Stimulator of interferon genes
NLRP3	NLR family pyrin domain containing 3
RME	Receptor-mediated endocytosis
CME	Clathrin-mediated endocytosis
CVME	Caveolin-mediated endocytosis
